# Worry and Permissive Parenting in Association with the Development of Internet Addiction in Children

**DOI:** 10.3390/ijerph17217722

**Published:** 2020-10-22

**Authors:** Barbara Chuen Yee Lo, Romance Nok Man Lai, Ting Kin Ng, Haobi Wang

**Affiliations:** 1 Department of Applied Psychology, Lingnan University, New Territories 100020, Hong Kong, China; nokmanlai@ln.hk; 2 Wofoo Joseph Lee Consulting and Counseling Psychology Research Centre, Lingnan University, Tuen Mun, New Territories 100020, Hong Kong, China; ngtingkin@gmail.com (T.K.N.); haobiwang@ln.edu.hk (H.W.)

**Keywords:** internet addiction, internet use, permissive parenting style, worry

## Abstract

The Internet has experienced a rapid increase in use globally. Specifically, more than 90% of Hong Kong’s citizens use the Internet, and 70% of children in the age group of 6–17 years have daily access to it. However, internet addiction could pose serious social and health issues. Therefore, conducting research to investigate its causes and risk factors is fundamental. The current study examined the relationship between worry and Internet addiction among children in Hong Kong and investigated the moderating effect of the permissive parenting style on such a relationship. The participants consisted of 227 fourth- and fifth-grade students (120 males, 52.9%) with a mean age of 9.55 (standard deviation (SD) = 0.58) in Hong Kong. Each participant was asked to complete the questionnaires, including the Internet Addiction Test for Internet addiction, the Penn State Worry Questionnaire for Children for worry, and the Parental Authority Questionnaire for the permissive parenting style. The results indicated that worry was related to greater Internet addiction among children. Furthermore, there was a moderating effect of the permissive parenting style such that the positive association between worry and Internet addiction was stronger when the permissive parenting style was higher. Our findings imply that parenting styles are influential in the prevention of Internet addiction.

## 1. Introduction

### 1.1. Internet Use among Children

With growing accessibility and need, the use of the Internet has increased rapidly over the past few decades [1]. With rapid and advanced development in information technology, more than 90% of the citizens in Hong Kong use the Internet [2], and 70% of children (aged 6 to 17 years) have daily access to it [3]. These statistics imply that Internet use has become essential specifically among the adolescent population. The recent COVID-19 pandemic and the preventive measures (e.g., suspensions of face-to-face classes, lockdown of shops and restaurants) has led to increased use of the Internet in the daily lives of children [4]. Therefore, Internet addiction has become an increasingly important public health issue worldwide.

### 1.2. Negative Impacts of Internet Addiction

Undoubtedly, the Internet provides various benefits to people’s daily lives. For example, the Internet is a tool for people to obtain information and knowledge [5]. Moreover, online social interactions might help to build social relationships [6]. However, Internet addiction could pose serious social and health issues, including related negative influences on one’s physical health, mental health, and social development [7,8]. According to the World Health Organization [9], Internet addiction is characterized by excessive or poorly controlled preoccupations, urges, or behaviors regarding internet use that lead to impairments or distress. This includes problematic Internet use, virtual addiction, pathological Internet use, pathological computer use, and compulsive Internet use. Empirical evidence has suggested an association between excessive use of the Internet and physical health problems such as obesity, vision problems, musculoskeletal problems, hearing impairments, and sleep deprivation [10,11,12,13,14]. Furthermore, more than four hours per day of screen-based activities (including the use of the Internet) could result in a higher risk of having somatic symptoms such as dizziness, headaches, tremors, or stomach aches [15]. Additionally, the authors pointed out that overuse of the Internet could cause numerous psychological distress symptoms, including feeling sad or hopeless [15]. Internet addiction may also lead to other poor mental health outcomes such as problematic substance use and suicidal ideation [5]. In sum, Internet addiction negatively affects addicts’ lives in numerous ways. Therefore, research on the risk factors for Internet addiction is of great importance.

### 1.3. Internet Addiction and Worry

Research on Internet addiction has demonstrated that worry is closely associated with Internet addiction and serves as a risk factor for Internet addiction [16]. Worry refers to an attempt to mentally problem-solve an issue whose outlook is uncertain and focuses on the possibility of negative outcomes [17]. Thus, worry is closely related to fear. Furthermore, the definition of worry has extended to an anxious apprehension for future negative events [18], which involves “a predominance of negatively valence verbal thought activity” and minimal levels of imagery [19]. Scholars have suggested that the experience of worry results from cognitive processes involved in anxiety, which function to maintain a great degree of vigilance for threat and danger [20]. Synchronously, worry is also a dominant feature of generalized anxiety disorder (GAD), and it could be reflected by apprehensive expectations regarding concerns in real-life (e.g., relations, finances, work, and school) [18,21].

Research has documented that negative emotions such as anxiety are related to addictive behaviors [22]. People who worry might adopt addictive behaviors, such as gambling, smoking and overuse of alcohol [23,24] as compensatory coping strategies to deal with negative emotions [23] or enhancing positive emotions [22]. Similarly, it is plausible that people who worry, who are characterized by having a relatively high intolerance for uncertainty, would suffer from the addictive use of smartphones and the Internet. There are several possible reasons for this. First, people who worry may constantly focus on uncertainties in their daily lives. From their perspective, the Internet could help them by providing various information resources, such as information surrounding health-related issues [25,26,27,28,29,30,31,32]. As a result, people with a high level of worry may excessively use the Internet to seek information and answers regarding their concerns [33]. People who worry usually experience ruminations and repetitive negative thinking when they face difficulties in life [16]. Since these obsessive thoughts are unpleasant experiences, they would like to find a way to stop them. Using smartphones or the Internet could be one of the strategies that help them distract or escape from negative feelings [34]. Therefore, when they experience challenges in life and suffer from many negative emotions, they would cope with them through compulsive Internet use. Briefly, people prone to worrying and those with a high intolerance of uncertainty would use the Internet more, and more easily suffer from Internet addiction.

### 1.4. The Role of Parents in Child Psychopathology and Internet Addiction

To prevent Internet addiction and the subsequent development of other psychopathologies among children, it is crucial to explore how parents influence children’s development, and how they can be helpful in risk identification in this context [35,36,37]. Many studies have indicated that parenting styles have important influences on child psychopathology and youth Internet dependency [35,38,39,40,41,42]. According to Buri [43] and Baumrind [44], parenting styles can be understood in terms of two dimensions, including parental demandingness or control and parental responsiveness or warmth. Parental demandingness refers to the extent to which a parent controls and monitors the child, whereas parental responsiveness refers to the extent to which a parent shows affective warmth, acceptance, nurturance and support to the child [43,44,45]. The two dimensions can be combined to form four parenting styles: (1) authoritative (high responsiveness and high demandingness), (2) authoritarian (low responsiveness and high demandingness), (3) permissive (high responsiveness and low demandingness), and (4) neglectful (low responsiveness and low demandingness).

Parenting styles that provide either no autonomy or unlimited autonomy for children would negatively influence children’s mental health. For example, children with controlling parents feel that they are not able to engage with the environment efficiently and independently. These children tend to perceive little control over their lives because their controlling parents typically do not involve them in decision making [46]. Hence, these children develop a low level of self-confidence, and cannot use effective emotional coping skills, leading to anxiety or other psychopathologies [44,47,48]. Besides, permissive parenting without being domineering would also result in childhood anxiety since there is insufficient guidance and direction for children to learn how to effectively manage their emotions [44,47].

Moreover, parenting styles are closely related to problematic behaviors among children, such as Internet addiction [49,50]. Recent research has showed that adolescents’ addictive Internet use is positively predicted by the permissive parenting style and negatively predicted by the authoritative parenting style [49,51]. It is argued that children with authoritative parents who have sufficient parental behavioral control will have a lower level of Internet addiction [39]. In contrast, due to a lack of expectations and monitoring, the permissive parenting style might increase the probability of Internet addiction among children [52]. Additionally, it is more likely that children who received permissive parenting will experience identity confusion, thereby enhancing the chance of Internet addiction [51]. These results reveal the importance of parenting styles on children’s addictive use of the Internet.

### 1.5. Permissive Parenting Style as a Potential Moderator

Although past research has demonstrated the significant role of parenting styles in children’s mental development, whether parenting styles can moderate the detrimental effect of worry on Internet addiction have not been well investigated [53,54]. A study found that the permissive parenting style moderated the relationship between sensation seeking and problematic internet use in adolescents [55], suggesting that it is possible for the permissive parenting style to moderate the effects of child characteristics on internet addiction.

Previous research has suggested that people who are prone to worrying are likely to develop problematic Internet use [34,56,57]. However, there are different levels of Internet use and addiction. Some children who worry use the Internet excessively but still have certain controls, while others with Internet addiction encounter numerous negative consequences in their daily lives [58]. There is a possibility that a maladaptive parenting style might be a moderator in this context that exacerbates the harmful effect of worry on Internet addiction.

As stated earlier, worrying children seek information to resolve uncertainty on the Internet, and use it as a tool to overcome negative thoughts. Under these circumstances, if parents provide more monitoring, children’s worry and negative emotions may not result in Internet addiction [50]. Conversely, if parents do not provide guidance and impose restraints, children’s worries will continue to recur, and they will have difficulties stopping anxiety-provoking compulsions and balancing the time spent on the Internet [50]. Additionally, the permissive parenting style provides too much freedom and too little advice and control on children, resulting in more self-doubts in children. This, in turn, makes them prone to becoming dependent on others, incapable of controlling their impulses, and unable to face complicated situations with confidence [59,60]. A vicious cycle may develop, in which anxious children seek assistance, reassurance, and support from surfing the Internet, finally resulting in compulsive Internet use [50].

**Figure 1:**
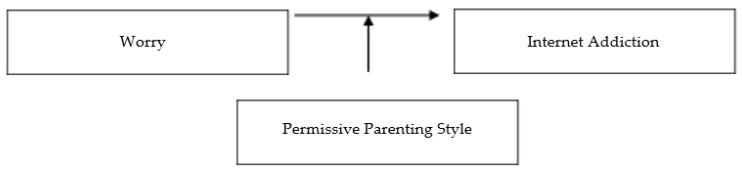
Hypothesized moderating effect of the permissive parenting style on the relationships between worry and Internet addiction.

In view of this, the present research aims to investigate the moderating effect of the permissive parenting style on the relationship between worry and Internet addiction among children. Based on the above theoretical framework and past studies [61], it is feasible that the permissive parenting style could moderate the relationship between children’s vulnerable traits and problematic behavioral outcomes. Specifically, for this study, it is hypothesized that the permissive parenting style will exacerbate the detrimental effect of worry on Internet addiction in children (Figure 1). Fourth- and fifth-grade primary school students in Hong Kong were investigated in this research with a cross-sectional design.

## 2. Methodology

### 2.1. Participants and Procedures

A total of 227 fourth- and fifth-grade children were recruited from primary schools in Hong Kong. There were 120 males (52.9%) and 107 females (47.1%). Their age ranged from 8 to 12 years (mean (M) = 9.55, standard deviation (SD) = 0.58). The research purpose was first explained to the school, and the researchers asked for approval to conduct the study. The questionnaires were then sent to the school and distributed to students by teachers. Participation was voluntary, and the participants were kept anonymous. The process took 20 min to complete, in which the questionnaires were distributed in the class under the supervision of trained teachers. All questionnaires are written in Chinese; hence, the participants were required to be capable of reading and understanding related Chinese words. The completed questionnaires were collected by the researchers at the end of the experimental session. This study received ethical approval from the Office of Research Support of Lingnan University (ethical code number: EC-007/1819) on 13 November 2018.

### 2.2. Measures

#### 2.2.1. Internet Addiction Test (IAT)

Internet addiction was measured using a Chinese version of the Internet Addiction Test (IAT). The IAT contains 20 items rated on a 5-point Likert scale (1 = not at all, 5 = always) [58]. It was developed based on the Young Diagnostic Questionnaire (YDQ), and was used to measure the level of Internet involvement and addiction from the perspective of one’s psychological symptoms and behaviors, including psychological dependence, compulsive use, withdrawal, problems with daily routine, sleeping pattern, family, social life, and time management. A higher score indicates greater problems due to Internet use. According to Young [58], individuals are placed into one of the three categories: average online users (from 20 to 39) who have complete control over the usage; persons who experience frequent problems due to excessive Internet use (from 40 to 69); and persons who have numerous problems because of Internet addiction (from 70 to 100). Previous studies [62,63,64,65] showed that that IAT possesses strong internal consistency and good test–retest reliability. The IAT also demonstrated a favorable internal consistency in a Hong Kong sample [66]. In this study, the IAT showed high internal consistency reliability (Cronbach’s α = 0.919).

#### 2.2.2. Penn State Worry Questionnaire for Children (PSWQ-C)

The level of worry was measured using the Chinese version of the Penn State Worry Questionnaire for Children (PSWQ-C) [67]. This scale consists of 14 items assessed on a 4-point Likert scale (0 = not at all typical of me, 3 = very typical of me) to assess worry in children aged 6 to 18 years. A sample item is, “Once I start worrying, I can’t stop”. The total scale score ranges from 0 to 42. Higher scores represented a greater tendency to worry. Past research has found that the PSWQ-C demonstrated strong reliability in terms of internal consistency and test–retest stability [67]. In this study, the PSWQ-C exhibited good internal consistency reliability (Cronbach’s α = 0.889).

#### 2.2.3. Parental Authority Questionnaire

Parenting style was measured using the Chinese short version of the Parental Authority Questionnaire (PAQ) [43]. The PAQ is a 30-item questionnaire based on Baumrind’s [44] model of parenting, assessing children’s perceptions of parental permissiveness, authoritarianism, and authoritativeness. The degree of agreement with the statements regarding both mothers’ and fathers’ parenting is rated on a five-point Likert scale (1 = strongly disagree, 5 = strongly agree). Due to the sole interest of permissive parenting in our research, only 10 items measuring the level of permissive parenting style were used. Sample items include: “As I was growing up, my parents seldom gave me expectations and guidelines for my behavior,” and “As I was growing up, my parents allowed me to decide most things for myself without a lot of direction from them.” In this study, the permissive parenting style subscale yielded an adequate internal consistency reliability (Cronbach’s α = 0.741).

### 2.3. Data Analysis

Data were analyzed using the SPSS software (IBM, New York, NY, United States). To test the moderation hypothesis, a moderated regression analysis was conducted using the SPSS Macro PROCESS [68] (Model 1). The predictor (worry) and moderator (permissive parenting style) were mean-centered. An interaction term was formed by multiplying the centered predictor and moderator.

## 3. Result

### 3.1. Preliminary Analyses

Fisher’s F tests were first adopted to explore the impacts of gender on study variables. As shown in Table 1, the results indicated that there were no significant differences between boys and girls in their levels of worry and Internet addiction as well as in their parents’ permissive parenting style.

### 3.2. Analyses of Moderation

**Figure 2:**
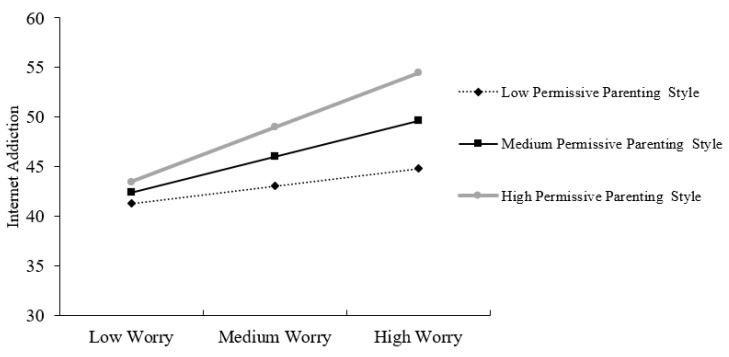
Simple slopes for the effect of worry on Internet addiction at low, medium, and high levels of the permissive parenting style.

Moderated regression analysis was conducted to examine whether the relationship between children’s worry and Internet addiction would be moderated by their parent’s permissive parenting style. As displayed in Table 2, the results found a significant children’s worry × permissive parenting style interaction effect on Internet addiction when controlling for children’s gender and age (unstandardized coefficient (*b*) = 0.029, *t*-test (*t*) = 2.115, *p =* 0.036). The interaction was plotted by substituting high (one standard deviation above the mean), medium (mean), and low (one standard deviation below the mean) values of worry and permissive parenting style into the regression equation (see Figure 2). The results of simple slope analyses showed that when permissive parenting style was low, the relationship between children’s worry and Internet addiction was non-significant (*b* = 0.188, *t* = 1.142, *p =* 0.255). When permissive parenting style was medium, children’s worry was significantly and positively related to Internet addiction (*b* = 0.388, *t* = 3.294, *p =* 0.001). When permissive parenting style was high, the relationship between children’s worry and Internet addiction was significant (*b* = 0.588, *t* = 4.301, *p* < 0.001).

## 4. Discussion

### 4.1. Discussion of Major Findings

In this study, we explored whether the permissive parenting style moderates the relationship between worry and Internet addiction among children. We found a moderating effect of the permissive parenting style on the relationship between worry and Internet addiction in primary school children. In particular, a high level of permissive parenting, characterized by high warmth but low parental monitoring, significantly enhanced the positive relationship between worry and Internet addiction in school-aged children.

This result could be explained by the distinct characteristics of school-aged children. In Erikson’s stages of psychosocial development [69], school-aged children are developing competence and thus it is necessary for them to gain new knowledge and skills, along with relative achievement and recognition from influential people such as parents and teachers. If they fail to achieve this, they may develop a sense of inferiority regarding their abilities and could feel uncertain about their future. During this period, children would experience many “first times”, such as presentation, school camp, and puberty’s physical and psychological changes, which create certain stresses [70]. Therefore, parents play a major role in helping their children in this stage to gain the necessary knowledge and skills and build self-confidence. To be a supportive parent, it is fundamental to consider children’s interests, to have reasonable expectations, and to provide encouragement, reinforcement, and some insights for problem solving [70]. However, some parents, especially those who are permissive, may provide care and love, but may not be able to assist their children by setting goals or teaching them the way to deal with their uncertainty and problems. Hence, it is also possible for school-aged children to develop worry or find answers on the Internet, especially for those who are prone to worrying and who are learning to be independent from their parents. Accordingly, these explanations provide possible support for the observed result that a high level of permissive parenting strengthens the relationship between worry and Internet addiction for school-aged children.

As reflected in past studies [71], parental monitoring is an important protective factor for Internet addiction, especially for senior high school students. The present results suggest that this effect could possibly extend to younger primary school children as well. Although permissive parents usually actively respond to children with love and warmth, there is a lack of monitoring, expectations, and given explanations, which may not help to address children’s concerns [59]. In this context, children may be prone to becoming doubtful, which may result in confusion in themselves [51], thus promoting Internet addiction. With the unlimited freedom given by the Internet, children are incapable of controlling themselves [60]. Consequently, when they face uncertainty and problems, they feel worried, and doubt whether their parents could help them solve the difficulties. This fosters the development of compulsive behaviors such as Internet addiction. With less parental monitoring or support, parents might also fail to set limits or rules on bedtimes and daily schedules of their children, and thus increase their risk for Internet addiction [72]. In general, a high level of permissive parenting without clear rules and effective monitoring would enhance the effect of worry on the development of Internet addiction.

Parental monitoring could be especially important for Chinese children. Chinese culture puts a great emphasis on parents’ responsibility in disciplining and monitoring their children [48]. With a lack of parental control, Chinese children may not feel loved by their parents. Chao [73,74] reported that instead of being permissive, being strict, controlling, and highly involved in children’s lives are key characteristics of traditional Chinese parenting practices. Compared with Western parents, Chinese parents tend to be more controlling and overinvolved [48]. More importantly, it was found that Chinese students linked the perception of parental psychological control with parental involvement [60,75]. This suggests that with lower parental control, children may think their parents are not involved. Additionally, Chinese parents usually exercise control, concern, and governance, rather than hugging or verbal expressions, to express their love, warmth, and support [73,74]. It is possible that the participants who were Chinese children with permissive parents perceived their parents as inattentive and neglectful. They may perceive that when their parents do not monitor and communicate their expectations, it indicates that their parents do not care about them. In view of this perspective, a high level of permissive parenting may not only be characterized by low control, but also could be interpreted as low responsiveness. Thus, these parents cannot help minimize the effect of worry on Internet addiction by implementing controls or emotional support.

### 4.2. Implications

Our findings reveal that the problematic use of technology might be one of the outcomes of a high level of worry, and, more importantly, permissive parenting can moderate the relationship between worry and Internet use. Supported by the analysis, parents should avoid implementing a high level of permissive parenting or insufficient monitoring regulations, in order to diminish the effect of children’s worry on problematic behaviors. As noted in both previous research and this research, school-aged children have many new experiences, including physical changes in the body and different events in school, which might negatively influence their emotions and cognitions [70]. By fourth or fifth grade, young children try to regulate their emotions and uncertainties by themselves [34,76,77]. Under these circumstances, the role of parents is important. If parents adopt a low level of permissive parenting with more guidance, education, and monitoring, their children will be more likely to manage negative thoughts with a clear and appropriate direction, instead of being confused and using the Internet to escape [50]. Parental warmth, support, advice, monitoring, and expectations could significantly influence the relationship between worry and Internet addiction among young children. Therefore, we suggest that parents pay attention to the psychological state of their children, understand the factors behind the psychopathology, and provide direction for children to cope with it. Accordingly, doing so will lead to a lower probability of children prone to worrying who develop Internet addiction.

Apart from parents’ parenting behaviors, the attitudes of parents and teachers are also important to reduce children’s Internet addiction. Parents’ and teachers’ non-permissive attitudes are related to less deviant and addictive behaviors among children [78]. Moreover, parents’ and teachers’ frequent, open, and positive communication patterns with children can prevent children from engaging in addictive behaviors [79]. As the recent COVID-19 pandemic has made children more dependent on the Internet in their daily lives, parents and teachers are suggested to involve children in more offline activities such as physical exercise to decrease their Internet use [4].

### 4.3. Limitations and Conclusions

The present study has several limitations. First, the cross-sectional design of this study precludes any causal inferences. In the future, it is important to apply a longitudinal design to further examine the relationship between worry and Internet addiction, along with the moderating effect of parenting style.

Second, our sample was limited to fourth- and fifth-grade students from primary schools in Hong Kong. As a result, the presented findings may not generalize to younger children, adolescents, adults, or children from different cultural backgrounds. As indicated by past research, children in different age groups may have different perceptions towards parents, and the parental influences for older children might not be as crucial as younger children, possibly driving differences in the observed patterns [80,81]. Future studies could seek to replicate the current findings using samples with a diverse range of child age and cultural backgrounds.

Third, the present study used child-reported data. It is possible that self-reporting bias would occur, although it is generally believed that children’s subjective experiences of parenting styles are more meaningful to their development than the objective measure of parenting patterns [82,83]. In future studies, one can consider other informant sources, and use multiple data collection methods to avoid method bias. For example, we can examine the effect of parenting using an observational measure [84] or an experimental approach [85]. Besides, future work may use a qualitative approach such as interviews to assess the parenting of Chinese-speaking parents in the Chinese cultural context [86]. By combining different kinds of measuring instruments, the validity and reliability of the research could be enhanced.

Lastly, additional variables could be collected for a more comprehensive assessment of Internet addiction. Children’s use of time for different activities on the Internet, the venue used to access the Internet, perceptions of Internet use, and examination of other parenting styles are some examples that could be included in corresponding studies. For example, Rozgonjuk’s [33] recent study considered smartphone use. Hence, future studies could discuss the relationship between intolerance of uncertainty and problematic smartphone use with more factual descriptions. In short, with detailed reported information from the participants, we could interpret our results with in-depth and more valid explanations in relation to the causes of Internet-addicted behaviors.

In conclusion, this purpose of this study was to examine the extent to which the association between worry and Internet addiction in children was moderated by the permissive parenting style. Consistent with our prediction, it was found that the relationship between worry and Internet addiction was significantly moderated by the permissive parenting style. More importantly, a high level of permissive parenting would exacerbate the detrimental influence of worry on Internet addiction among children in Hong Kong. It is suggested that parents of worried children should exert monitoring efforts in order to prevent their children from engaging in addictive Internet use.

## Figures and Tables

**Table 1:** Gender differences in worry, the permissive parenting style, and Internet addiction. M: mean; SD: standard deviation.

Variable	Overall		Boys		Girls		F
	M	SD	M	SD	M	SD	
Worry	17.921	9.339	17.592	9.033	18.290	9.700	0.315
Permissive parenting style	29.427	6.999	30.142	7.308	28.626	6.578	2.671
Internet addiction	46.560	16.516	47.883	16.892	45.075	16.033	1.640

Note: none of the gender differences was significant. M = mean; SD = standard deviation; F = F-test.

**Table 2:** The moderating effect of the permissive parenting style on the relationship between worry and Internet addiction.

Variable	*b*	*SE*	*β*	*t*	*p*
Age	3.824	1.782	0.232	2.146	0.033
Gender	−2.727	2.045	−0.165	−1.333	0.184
Worry	0.388	0.118	0.179	3.294	0.001
Permissive parenting style	0.422	0.154	0.219	2.742	0.007
Worry × permissive parenting style	0.029	0.014	0.113	2.115	0.036

Note: *b* = unstandardized coefficient; *SE* = standard error; *β* = standardized coefficient Beta; t = *t*-test.
